# Hop-Derived Iso-α-Acids in Beer Improve Visual Discrimination and Reversal Learning in Mice as Assessed by a Touch Panel Operant System

**DOI:** 10.3389/fnbeh.2019.00067

**Published:** 2019-04-02

**Authors:** Tatsuhiro Ayabe, Rena Ohya, Yasuhisa Ano

**Affiliations:** Research Laboratories for Health Science & Food Technologies, Kirin Company Ltd., Yokohama, Japan

**Keywords:** iso-α-acids, touch panel operant system, visual discrimination, reversal learning, dopamine

## Abstract

Dementia and cognitive decline have become worldwide health problems due to rapid growth of the aged population in many countries. We previously demonstrated that single or short-term administration of iso-α-acids, hop-derived bitter acids in beer, improves the spatial memory of scopolamine-induced amnesia model mice in the Y-maze and enhances novel object recognition in normal mice *via* activation of the vagus nerve and hippocampal dopaminergic system. However, these behavioral tests do not replicate the stimulus conditions or response requirements of human memory tests, and so may have poor translational validity. In this report, we investigated the effects of iso-α-acids on visual discrimination (VD) and reversal discrimination (RD) using a touch panel-based operant system similar to that used for human working memory tests. In the VD task, scopolamine treatment reduced correct response rate and prolonged response latency in mice, deficits reversed by prior oral administration of iso-α-acids. In the RD task, administration of iso-α-acids significantly increased correct response rate compared to vehicle administration. Previous studies have reported that dopamine signaling is involved in both VD and RD learning, suggesting that enhancement of dopamine release contributes to improved memory performance in mice treated with iso-α-acids. Taken together, iso-α-acids improve VD and RD learning, which are considered high-order cognitive functions. Given the translational advantages of the touch panel-based operant system, the present study suggests that iso-α-acids could be effective for improvement of working memory in human dementia patients.

## Introduction

Dementia and age-related cognitive decline are increasing in prevalence as our populations age. Effective therapies for dementia after onset have not been established, so preventive strategies such as exercise and improved dietary habits have drawn increasing attention. A meta-analysis concluded that consumption of low to moderate amounts of alcoholic beverages may reduce the risk for dementia (Neafsey and Collins, 2011). While this effect may depend on alcohol itself, several compounds contained in alcoholic beverages are reported to have neuroprotective properties. Resveratrol for example, a polyphenolic compound contained in red wine, has been reported to improve cognitive function in dementia model rodents (Huang et al., 2011; Ma et al., 2013) and healthy adults (Kennedy et al., 2010). Our group found that long-term intake of iso-α-acids, bitter acids in beer derived from hops (*Humulus lupulus L*.), prevents memory impairment in Alzheimer’s disease (AD) model mice (Ano et al., 2017) and high fat diet-induced obese mice (Ayabe et al., 2018a). We also reported that single or short-term administration of iso-α-acids increases dopamine levels in the hippocampus *via* vagus nerve activation (Ano et al., 2019). Further, oral administration of iso-α-acids improves short-term spatial memory in the Y-maze test and object recognition memory in the novel object recognition test. While these simple behavioral procedures are suitable for drug screening (Yamada et al., 2010; Ano et al., 2018; Ayabe et al., 2018b), the perceptual and behavioral requirements are vastly different from the cognitive assessments used to test working memory in humans.

Recently, touch panel-based operant systems have been developed to assess higher-order brain functions in rodents. The touch panel operant systems are established based on Pavlovian autoshaping or sign- and goal-tracking (Parkinson et al., 2000b; Tomie et al., 2003; Lopez et al., 2015), and are related to various brain regions including cingulate and medial prefrontal cortex (Bussey et al., 1997a,b), amygdala (Parkinson et al., 2000a), nucleus accumbens (Parkinson et al., 2000b), and hippocampus (Ito et al., 2005). Since the touch panel operant systems are associated with various cognitive and psychiatric functions, they have been used for researches on AD (Romberg et al., 2013), schizophrenia (Brigman et al., 2006, 2009), and Huntington’s disease (Morton et al., 2006). These systems allow researchers to perform more translatable tests in rodents because of their similarity to human cognitive tests, such as the Cambridge Neuropsychological Test Automated Battery (CANTAB; Barnett et al., 2016). Indeed, Nithianantharajah et al. (2015) demonstrated that mice and humans carrying the same disease-related genetic mutations exhibited similar cognitive impairment in paired associates learning (PAL) test paradigms. Previous studies have reported that hippocampal catecholamines, including dopamine, are involved in PAL task performance (Talpos et al., 2015; Roschlau and Hauber, 2017). Using touch panel operant systems, it may be possible to evaluate the cognitive benefits of iso-α-acids and elucidate the underlying mechanisms in rodents with high translational validity.

Touch panel operant systems are well suited for analysis of visual discrimination (VD) and reversal discrimination (RD; Oomen et al., 2013; Morita et al., 2016; Piiponniemi et al., 2017). The VD task requires the integration of perceptual learning and memory processing (Gilbert et al., 2001; Bussey and Saksida, 2007), while the RD task requires flexibility of memory function (Kehagia et al., 2010; Klanker et al., 2013). Impairments of VD and RD learning are observed in patients with AD (Freedman and Oscar-Berman, 1989), AD model mice (Romberg et al., 2013; Piiponniemi et al., 2017), and scopolamine-induced amnesia model mice, which also exhibit AD-like pathology (Winters et al., 2010). Further, monoamine neurotransmitters including dopamine are involved in VD and RD learning tasks (Haber, 2014; Morita et al., 2016; Takaji et al., 2016). Therefore, iso-α-acids, which increase dopamine release, are expected to improve VD and RD task performance. In the present study, we investigated the effects of short-term iso-α-acids administration on VD and RD learning in amnesic and control mice using a touch panel operant system.

## Materials and Methods

### Materials

We used isomerized hop extract (IHE) as a source of iso-α-acids. IHE was purchased from Hopsteiner (Mainburg, Germany) as a potassium salt in aqueous solution. The contents of this IHE were analyzed and described previously (Ano et al., 2017). Briefly, this IHE contains 30.5% (w/v) iso-α-acids, comprised of trans-isocohumulone (1.74% w/v), cis-isocohumulone (7.61% w/v), trans-isohumulone (3.05% w/v), cis-isohumulone (14.0% w/v), trans-isoadhumulone (0.737% w/v), and cis-isoadhumulone (3.37% w/v). IHE also contains components other than iso-α-acids, including low levels of α-acids (<0.6%), β-acids (<0.2%), and hop oil (<0.1%). Scopolamine was purchased from Sigma Aldrich Company (St. Louis, MO, USA).

### Animals

Seven-week-old male C57BL/6J mice were purchased from Charles River Japan Inc. (Tokyo, Japan). Mice were housed at room temperature (23 ± 1°C) under a constant 12-h/12-h light/dark cycle (light period from 8:00 am to 8:00 pm) and fed standard rodent chow (CE-2, Clea Japan, Tokyo, Japan) for 1 week prior to experiments. In total, 50 mice were utilized. All animal care and experimental procedures were performed according to the guidelines of the Animal Experiment Committee of Kirin Company Ltd., and all efforts were made to minimize suffering. All studies were approved by the Animal Experiment Committee of Kirin Company Limited and conducted in 2017 (approval IDs AN10163-Z00 and AN10364-Z00).

### Touch Panel Operant Test

#### Apparatus

**Figure 1:**
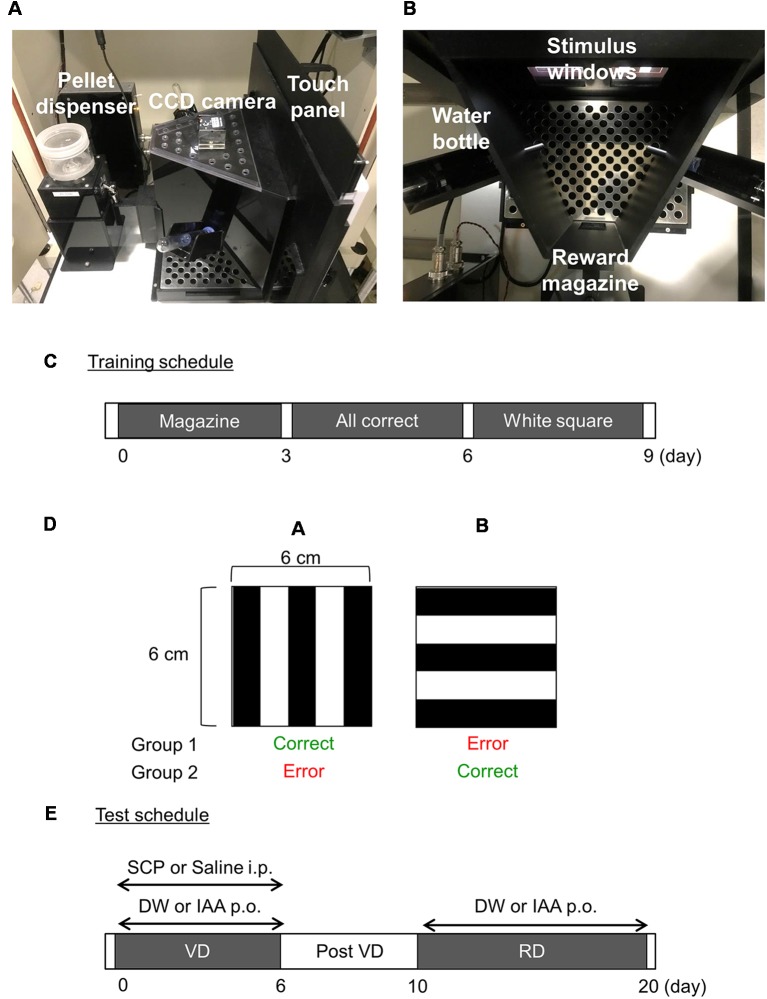
The touch panel-based operant system and testing procedures. **(A,B)** The touch panel operant system apparatus. The apparatus is composed of a touch panel, pellet dispenser, and water bottles. The apparatus is contained within a sound-isolated chamber. A CCD camera is mounted on the apparatus. The touch panel is divided into two stimulus windows by a black wooden board, and a reward magazine is placed at the opposite side of the touch panel. **(C)** Training schedule. **(D)** Vertical and horizontal stripes were used as stimuli in visual discrimination (VD) and reversal discrimination (RD) tasks. **(E)** Testing schedule. SCP, scopolamine; DW, distilled water; IAA, iso-α-acids.

The touch panel operant test apparatus (O’HARA & Co., Ltd., Tokyo, Japan) consists of a touch panel, a pellet dispenser delivering 10-mg food pellets (AIN-76A Rodent Tablet; TestDiet, St. Louis, MO, USA), and water bottles (Figure 1A). The touch panel was divided into two stimulus windows (each 6 cm × 6 cm) by a black wooden board. The pellet dispenser or reward magazine was placed at the opposite side of the touch panel (Figure 1B). A CCD camera was also mounted on the apparatus. For experiments, the apparatus was enclosed in a sound-isolated chamber with a house lamp and an audio speaker. All experiments were conducted in a sound-isolated room (23 ± 1°C).

#### Animal Habituation

Prior to experiments, feeding was restricted to reduce body weight to around 80% of age-matched *ad lib* fed-mice. Body weight was measured as frequently as possible to avoid excessive body weight reduction. After body weight reached the 80% criterion, mice were acclimated to the food pellets (rewards) by feeding each animal 100 pellets per day for 3 days. Mice were then placed in the touch panel operant test apparatus for 15 min per day for 3 days without any task requirements. During this period, 15 reward pellets were placed in the dispenser (reward magazine) so that the mouse learned to associate this location with food rewards.

#### Pre-training

The pre-training phase consisted of three periods (Figure 1C): (i) Magazine training; (ii) All correct training; and (iii) White square training. (i) For Magazine training, reward pellets were automatically delivered every 1 min for 15 min accompanied by a tone so that the mouse learned that the tone indicated reward presentation in the dispenser. Magazine training was conducted once daily for 3 days. (ii) During All correct training, white squares were presented on both stimulus windows, and reward pellets were delivered when the mouse touched either window. Each task session lasted 50 trials (stimulus-reward pairings) or 15 min, whichever came first. This training was conducted daily for 3 days. (iii) During White square training, a white square was presented randomly on one stimulus window, while the other window remained blank. Reward pellets were delivered with a reward tone when the mouse touched the window presenting the white square. Again, sessions lasted 50 trials or 15 min, whichever came first, and were conducted daily for 3 days.

#### Visual Discrimination Task

In the VD task, a pair of visual stimuli (vertical and horizontal stripes) appeared on the screen during each trial. Half the mice were presented the vertical stripes as the correct (rewarded) response and the horizontal stripes as the incorrect response, while the condition was reversed for the remaining mice (Figure 1D). A trial started when the mouse touched the reward magazine. A nose poke to the correct stimulus resulted in tone and reward delivery, followed by a 2-s inter-trial interval (ITI). A nose poke to the incorrect stimulus resulted in no reward, 5 s of darkness (lights out), and a 5-s ITI. After each ITI, the next trial started when the mouse touched the reward magazine. A trial was omitted when the mouse did not touch either stimulus within 30 s. Iso-α-acids solution (1 mg/kg body weight) or distilled water (DW) was administered by oral gavage 60 min before the test session and scopolamine (0.8 mg/kg body weight) or saline was intraperitoneally administered 30 min before the test session (Figure 1E).

#### Reversal Discrimination Task

Both the scopolamine-treated group and iso-α-acid-treated group then performed the RD task. Before starting the RD task, mice continued performing the VD task without drug treatment until a criterion correct responses rate >80% was reached (post-VD training). In the RD task, the correct and incorrect stimuli were reversed relative to the VD session. For example, mice presented vertical stripes as the correct stimulus in the VD task were presented horizontal stripes as the correct stimulus in the RD task. Again, iso-α-acids solution (1 mg/kg body weight) or DW was administered by oral gavage 60 min before the test. Mice were not treated with scopolamine in the RD task. In total, DW/IAA was injected 17 times, and scopolamine was injected seven times, in all experiment period. The number of correct response changes (Δ Correct response rate) was calculated as (Correct response rate of each daily trial) − (Correct response rate of 1st trial) to evaluate how effectively the mice could change their previous memory conditions.

### Statistical Analysis

**Figure 2:**
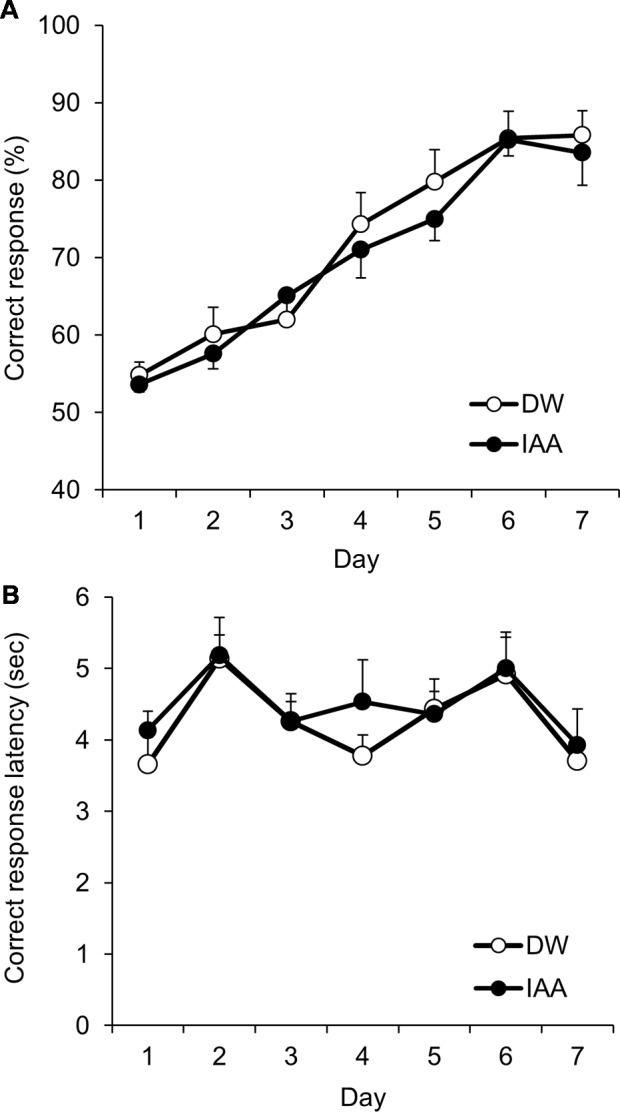
VD learning in untreated control mice. **(A)** Proportion of correct responses (%) across sessions by DW-treated mice (control group) and iso-α-acids-treated mice (IAA group) in the VD task. **(B)** Correct response latency, which indicates the time between the trial start and correct response. All values are expressed as mean ± SEM. Control group: *n* = 10; IAA group: *n* = 10.

All values are expressed as mean ± SEM. Correct response rates of untreated mice (Figure 2) were analyzed by one-way repeated-measures ANOVA followed by the Bonferroni’s test. Correct response rates and response delays of treated mice during the VD and RD tasks were analyzed by two-way repeated-measures ANOVA followed by Bonferroni’s test or student’s *t*-test. For data without repeated measures, paired groups were compared by student’s *t*-test and multiple groups by one-way ANOVA followed by Tukey–Kramer tests for pair-wise comparisons. All statistical analyses were performed using the Ekuseru-Toukei 2012 software package (Social Survey Research Information, Tokyo, Japan). A *P* < 0.05 (two-tailed) was considered statistically significant for all tests.

## Results

### Confirmation of Visual Discrimination Learning in the Touch Panel Operant System

Before using the scopolamine-induced amnesia model mice, we first confirmed the acquisition of VD learning and examined the effect of iso-α-acids in the touch panel operant system using normal untreated mice. A significant main effect of session days (*F*_(6,102)_ = 39.38; *P* < 0.001) with no significant main effect of treatment (*F*_(1,17)_ = 0.173, *P* = 0.683) and significant interaction (*F*_(6,102)_ = 0.535, *P* = 0.781) was noted. Correct response rate was significantly increased after the fourth daily session compared to day 1, and exceeded 85% on day 6 in both the groups. The treatment of iso-α-acids did not affect the VD learning as compared with that in the control mice (Figure 2A). Correct response latency, which is associated with attention for task stimuli, indicates significant main effect of the session days (*F*_(6,102)_ = 5.055; *P* < 0.001), with no significant main effect of the treatment (*F*_(1,17)_ = 0.210, *P* = 0.653) and significant interaction (*F*_(6,102)_ = 0.462, *P* = 0.835; Figure 2B). These results indicate that normal mice successfully learned the VD task using the touch panel operant system.

### Oral Administration of Iso-α-Acids Improves Visual Discrimination Learning of Scopolamine-Induced Amnesia Model Mice

**Figure 3:**
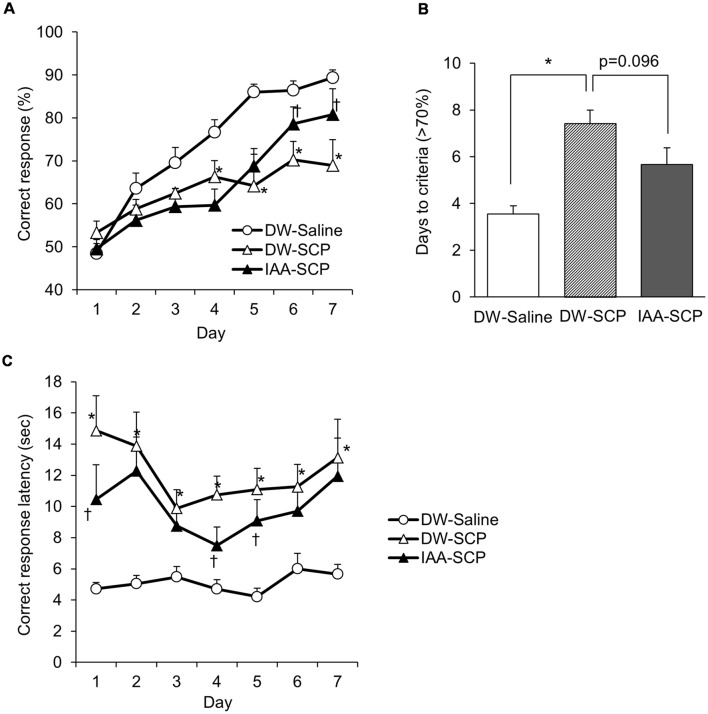
Iso-α-acids improve VD impairments in scopolamine-induced amnesia model mice. **(A)** Correct responses (%) across sessions in the VD task. Mice were divided into three groups: DW and saline-treated group (DW-saline group), DW and scopolamine-treated group (DW-SCP group), and iso-α-acids and scopolamine-treated group (IAA-SCP group). Mice were administered DW or iso-α-acids (1 mg/kg) by oral gavage 60 min before the task and intraperitoneally injected with saline or scopolamine (0.8 mg/kg) 30 min before the task. **(B)** Daily sessions required to reach the correct response criterion (>70%). **(C)** Correct response latency. All values are expressed as mean ± SEM DW-saline group: *n* = 9; DW-SCP group: *n* = 7; IAA-SCP group: *n* = 6. *P < 0.05 vs. DW-saline group, ^†^*P* < 0.05 vs. DW-SCP group.

Using the touch panel-based operant system, we evaluated VD learning by scopolamine-induced amnesia model mice and the potential benefits of iso-α-acids. A total of 30 mice (10 mice/group) were subjected to the test, and 23 mice (DW-saline group: *n* = 9; DW-SCP group: *n* = 8; IAA-SCP group: *n* = 6) completed the VD task. The significant main effect of session days (*F*_(6,114)_ = 26.36; *P* < 0.001) and treatment (*F*_(2,19)_ = 11.35; *P* < 0.001), and a significant session days × treatment interaction (*F*_(12,114)_ = 2.47; *P* = 0.006) was recorded. Compared to the DW-saline group, DW-SCP group demonstrated a significantly lower correct response rate after four daily VD learning sessions, while oral administration of iso-α-acids prior to scopolamine reversed this effect, resulting in significantly increased correct response rates on days 6 and 7 compared to mice treated with DW and scopolamine (Figure 3A). The number of daily sessions required to reach a >70% correct response rate was also significantly greater in DW and scopolamine-treated mice compared to DW and saline-treated mice, and this effect as well was reversed by iso-α-acids (*F*_(2,19)_ = 14.47; *P* < 0.001; Figure 3B). Thus, scopolamine treatment impairs VD learning, while oral administration of iso-α-acids improves VD learning of scopolamine-induced amnesia model mice. In addition to impaired correct response rate, correct response latency revealed a significant main effect of the session days (*F*_(6,114)_ = 2.83; *P* = 0.013) and treatment (*F*_(2,19)_ = 24.38; *P* < 0.001), albeit no significant interaction (*F*_(12,114)_ = 1.04, *P* = 0.41). The comparison of each session day showed a significant delay in DW and scopolamine-treated mice when compared with that in DW and saline-treated mice on all experimental days, while iso-α-acid treatment significantly shortened this latency when compared with that in DW and scopolamine-treated mice on days 1, 4, and 5 (Figure 3C). These results indicate that scopolamine treatment also impairs attention, a deficit reversed by administration of iso-α-acids.

### Oral Administration of Iso-α-Acids Improves Reversal Learning

**Figure 4:**
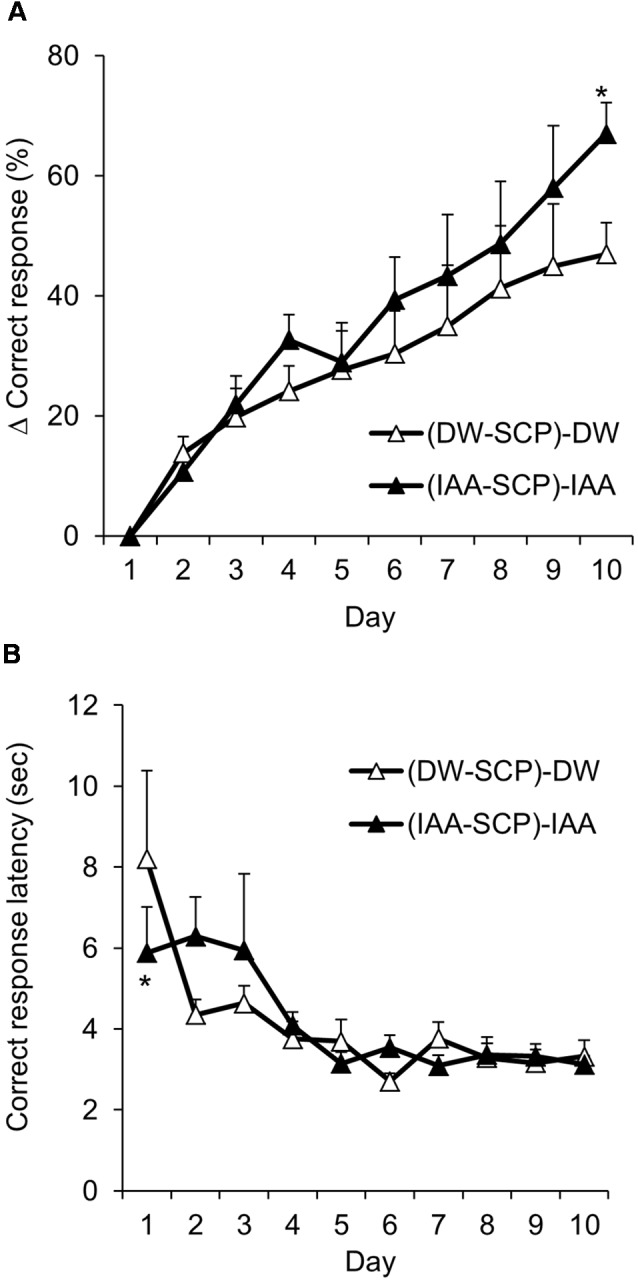
Iso-α-acids improve reversal learning. Mice from the DW-SCP group and the IAA-SCP group in the VD task were further treated with DW [(DW-SCP)-DW group] or iso-α-acids [(IAA-SCP)-IAA group], respectively, and subjected to RD task. **(A)** Changes in correct response rate across sessions in the RD task Δ correct response was calculated as (Correct response rate of each daily trial) – (Correct response rate of 1st trial). Mice were administered DW or iso-α-acids (1 mg/kg) by oral gavage 60 min before the task. **(B)** Correct response latency. All values are expressed as mean ± SEM. (DW-SCP)-DW group: *n* = 7; (IAA-SCP)-IAA group: *n* = 6. *P < 0.05 vs. control group.

To further examine the effects of iso-α-acids on higher-order cognitive functions, we tested mice in the RD task. DW and scopolamine-treated mice and iso-α-acids and scopolamine-treated mice were first subjected to additional VD task training sessions without further drug treatment. The correct and incorrect responses were then reversed for the subsequent RD sessions. The DW-SCP group and IAA-SCP group were again treated with DW and iso-α-acids, respectively, but not with scopolamine [(DW-SCP)-DW group and (IAA-SCP)-IAA group]. Neither the DW-saline group nor the IAA-saline group was subjected to the RD task. There was a significant main effect of session days (*F*_(9,99)_ = 38.30; *P* < 0.001), but no significant main effect of the treatment (*F*_(1,11)_ = 1.220; *P* < 0.293) and no significant interaction (*F*_(9,99)_ = 1.444; *P* = 0.180) were noted. Comparison of each session days revealed that the oral administration of iso-α-acids significantly increased the Δ correct response rate compared to control treatment on day 10 (*F*_(1,33)_ = 6.115; *P* < 0.019; Figure 4A), indicating that iso-α-acids can enhance cognitive flexibility. In the correct response latency, there was a significant main effect of session days (*F*_(9,99)_ = 6.272; *P* < 0.001), but no significant main effect of the treatment (*F*_(1,11)_ = 0.029; *P* = 0.866) and no significant interaction (*F*_(9,99)_ = 1.248; *P* = 0.275) were noted. In each session, correct response latency was significantly shortened by iso-α-acids treatment on day 1 but not on subsequent training days (Figure 4B). Thus, iso-α-acids may also enhance attention, but this effect was not as potent as observed in DW and scopolamine-treated mice in VD task.

## Discussion

Administration of iso-α-acids improved both VD learning and reversal learning in mice as measured using a touch panel-based operant system. We previously reported that iso-α-acids improve spatial and object memory in the Y-maze and the novel object recognition test, respectively, which are tests based on the curiosity of rodents for novel places and objects. While the Y-maze test and the novel object recognition test are well-established procedures to evaluate the effects of drugs and food components on memory (Yamada et al., 2010; Ano et al., 2018; Ayabe et al., 2018b), they do not engage the same cognitive and behavioral processes required for performance in tasks commonly employed to test human learning and memory. In contrast, the touch panel-based operant test has high translational potential because it resembles human touch panel-based cognitive measurement paradigms such as the CANTAB. Indeed, mice and humans carrying the same disease-related mutations exhibited similar cognitive impairments in a touch panel-based cognitive test paradigm (Nithianantharajah et al., 2015). In the present study, we found that iso-α-acids at a dose of 1 mg/kg improved VD and RD as measured using a touch panel-based operant test. Although we have not yet conducted clinical trials on iso-α-acids, these results strongly suggest potential cognitive benefits for human dementia patients. The effective dose of iso-α-acids in mice (1 mg/kg) is equivalent to only 4.8 mg/day in humans (60 kg body weight) according to the human equivalent dose modulus (0.08). In our previous study, some major types of beer (e.g., the lager type) contained 16–27 mg/L of iso-α-acids (Taniguchi et al., 2015). Based on these results, it is expected that intake of iso-α-acids at 4.8 mg/day or 0.17–0.3 L/day of beers for several weeks will improve VD and RD in cognitive tests such as the CANTAB.

In addition to the short-term effect of iso-α-acids on the improvement of cognitive function *via* dopamine signaling, we have previously reported that long-term administration of iso-α-acids prevents cognitive impairment in AD model mice *via* modulation of microglia (Ano et al., 2017). There may be some long-term effects of iso-α-acids on the VD task, and the lingering effect of previous iso-α-acid treatment in the RD task. However, to the best of our knowledge, modulation of the microglia does not directly affect VD and reversal learning. Although it is difficult to completely exclude the long-term effects, the accumulation of the short-term effect of iso-α-acids could be responsible for the improvement of VD and reversal learning in this study. Further studies, such as those assessing the performance of the RD task by intact mice without any previous treatment, are required.

VD learning requires integration of perceptual learning and memory, while reversal learning requires cognitive flexibility, and both are considered higher-order cognitive functions (Gilbert et al., 2001; Bussey and Saksida, 2007; Kehagia et al., 2010; Klanker et al., 2013). The precise mechanisms underlying VD and RD are still unclear, but monoamine neurotransmitter signaling is likely critical. Morita et al. (2016) reported that the dopamine D2-like receptor is required for both VD and RD learning. Moreover, a recent clinical trial found that vagus nerve stimulation improved working memory performance and visual attention on computer-based cognitive tests (Sun et al., 2017), and we previously reported that single oral administration of iso-α-acids enhances dopamine release in the hippocampus *via* vagus nerve activation (Ano et al., 2019). Though dopamine signaling was not assessed in the current study, improvement of VD and reversal learning suggest enhancement of vagus nerve activity and hippocampal dopamine signaling by iso-α-acids treatment. Future studies using dopamine receptor antagonists or vagotomized mice are warranted to evaluate this possible mechanism. In addition to the dopamine signaling system, other neurotransmitters, such as serotonin and norepinephrine, are also involved in VD and reversal learning (Ward et al., 1999; Seu et al., 2009; Izquierdo et al., 2017). The involvement of these neurotransmitters could not be excluded from the present results. The hippocampus is essential for working memory as assessed by touch panel operant systems (Talpos et al., 2008, 2009; Abela et al., 2013). In addition, however, various regions of cerebral cortex are involved in human working memory (D’Esposito and Postle, 2015), so additional studies are needed to elucidate the effects of iso-α-acids on these regions.

Impairments in VD learning have been observed in several neurodegenerative diseases, including AD (Freedman and Oscar-Berman, 1989). In the present study, we used scopolamine-induced amnesia model mice as these animals exhibit AD-like cognitive impairments. Scopolamine is a muscarinic antagonist that temporarily impairs learning and memory function by inhibiting cholinergic neuronal systems, and is used widely to screen drugs for effects on dementia and cognitive decline (Yamada et al., 2010). Consistent with previous reports (Winters et al., 2010; Talpos et al., 2012), scopolamine-induced VD learning impairments in the touch panel-based operant test, while short-term administration of iso-α-acids improved VD learning in these amnesic mice by modulating the dopamine signaling system. To our knowledge, this is the first report to demonstrate improved VD learning in amnesia model mice using a touch panel operant system. Further studies of AD model mice are warranted to provide further evidence that short-term administration of iso-α-acids can improve the cognitive impairments of dementia.

In addition to AD, VD and reversal learning deficits are also major symptoms of schizophrenia that are associated with the impairment of hippocampal functions (Martinelli and Shergill, 2015; Reddy et al., 2016). The touch panel operant systems have been used to evaluate the VD and reversal learning impairment in rodent schizophrenia models as well as to investigate the molecular mechanisms underlying the onset of this disease (Alsio et al., 2015; Gould et al., 2015; Zeleznikow-Johnston et al., 2018). Findings of the present study demonstrated that the touch panel operant systems can be applied to evaluate the drug efficacy, making it possible to screen drugs for schizophrenia and other cognitive and psychiatric disorders using this method. The present results are insufficient to discuss the effects of iso-α-acids on schizophrenia-related cognitive impairment, but there is a possibility that iso-α-acids can be effective in various cognitive and psychiatric disorders.

We also demonstrated that administration of iso-α-acids can reduce correct response latency in scopolamine-treated mice. Though the VD task used in the present study is not specifically designed to assess attention, this effect suggests that iso-α-acids improve scopolamine-induced attention deficits. Iso-α-acids also shortened the response latency in day 1 of RD task, but not on the subsequent days. The effect of iso-α-acids on attention may be exhibited when there are some loads for attention, such as scopolamine treatment or changing the operant conditioning. Attention deficits are induced by cholinergic neuronal loss in animals (Voytko et al., 1994; Bucci et al., 1998), and are observed in AD patients (Ferreira-Vieira et al., 2016). In addition, dopamine is regarded as the major therapeutic target for attention-deficit hyperactivity disorder (ADHD) patients (Tarver et al., 2014). Modulation of dopamine signaling by iso-α-acids may thus be useful for the treatment of psychological disorders linked to dopaminergic system dysfunction in addition to cognitive disorders.

In conclusion, we evaluated the effects of iso-α-acids, hop-derived bitter acids in beer, on VD and RD learning by scopolamine-treated amnesic mice using a touch panel-based operate system to assess the potential of these compounds for dementia treatment. Indeed, iso-α-acids enhanced both VD and RD, suggesting improvements in perceptual learning, memory, and cognitive flexibility. Intake of iso-α-acids could be effective for improving higher-order cognitive functions in humans.

## Data Availability

The datasets generated for this study are available on request to the corresponding author.

## Ethics Statement

All animal care and experimental procedures were performed according to the guidelines of the Animal Experiment Committee of Kirin Company Ltd., and all efforts were made to minimize suffering. All studies were approved by the Animal Experiment Committee of Kirin Company Limited and conducted in 2017 (approval IDs AN10163-Z00 and AN10364-Z00).

## Author Contributions

TA and YA designed the most of experiments. TA and RO performed the experiments and analyzed the data. TA wrote the most of article. YA conceived and supervised the article.

## Conflict of Interest Statement

All authors (TA, RO and YA) are employed by Kirin Co. Ltd.
